# A study protocol “saving futures: developing an integrated model of rehabilitation and paediatric HIV care to foster success at school”

**DOI:** 10.1186/s40814-018-0372-7

**Published:** 2018-11-28

**Authors:** Verusia Chetty, Stacy Maddocks, Saul Cobbing, Jill Hanass-Hancock

**Affiliations:** 1 Discipline of Physiotherapy, School of Health Sciences, University of KwaZulu-Natal, Private Bag X54001, Durban, 4000 South Africa; 2 School of Health Sciences, University of KwaZulu-Natal, Private Bag X54001, Durban, 4000 South Africa; 3 South African Medical Research Council, Westville Durban, South Africa

**Keywords:** HIV, Paediatrics, Disability, Rehabilitation, South Africa

## Abstract

**Background:**

A significant number of children experience disabilities as a result of living with HIV, including those on antiretroviral therapy (ART). Current paediatric HIV care does not prioritise rehabilitation. Furthermore, little attention is paid to cognitive development and educational needs, thereby placing the future of these children at risk. This can be mitigated by providing rehabilitation services to help overcome these disabilities.

**Methods:**

The study will assess the feasibility (acceptability, practicality, preliminary efficacy) of an integrated model of rehabilitation and paediatric HIV care in order to improve diagnosis and interventions for disability amongst children living with HIV between the ages of 5 and 10 years. The model will integrate data entry and management tools, improving identification, referral, and linkage to care, with an intervention approach that can be used by trained lay health professionals. The study targets both physical and cognitive impairments that lead to disabilities to improve school readiness and success. Phase 1 will inform the design of an optimal integrated model of rehabilitation and paediatric HIV care in a public healthcare setting in South Africa. The study will first undertake a formative investigation of the factors impacting integration of rehabilitation with paediatric HIV care from the perspective of caregivers and health professionals. It will use qualitative methods, including in-depth interviews and focus group discussions. The knowledge from this phase will inform the design of the model in phase 2, and phase 3 will pilot the integrated rehabilitation and paediatric HIV model with the aim to improve school readiness for the participants at the study site. The pilot intervention will be formally evaluated.

**Discussion:**

The results from this study will determine whether the model has potential for widespread application in South African paediatric HIV care and recommend further possible modifications. This will inform the development of a proposal to support the current government initiative to strengthen disability and rehabilitation services. The study results will also inform South Africa’s current efforts to strengthen early interventions for children with disabilities and will be an important and critically needed step in the use of rehabilitation to strengthen paediatric HIV care in the region.

## Background

Prevention of mother to child transmission and anti-retroviral therapy (ART) in resource-poor settings has led to considerable decreases in HIV infections amongst children [1]. Nevertheless, 3.2 million children are living with HIV worldwide, 2.9 million (91%) of whom live in sub-Saharan Africa [2]. Now that access to ART has dramatically improved the life expectancies of children living with HIV, their quality of life, emotional well-being, day-to-day functioning, and school readiness/success are of even greater importance. However, a long life with chronic HIV comes with new health-related needs and for some, the risk of disability. This requires a shift in thinking towards a comprehensive continuum of care that integrates rehabilitation in managing paediatric HIV especially in the sub-Saharan African region where the development of these services lags far behind that of adults [1, 3–5].

Recent research in sub-Saharan Africa suggests that a large number (33–60%) of children living with HIV, including those on ART, experience diverse forms of disability during their childhood with language/speech, cognition, and motor development being most frequently reported [3, 5–7]. A recent meta-analysis found that working memory, executive functioning, and processing speed are the cognitive domains most affected in children and adolescents living with HIV [8]. Sherr et al. suggested that since the evidence for HIV-associated cognitive impairment is consistent, children living with HIV should be routinely and regularly monitored for developmental delays and cognitive dysfunction so they can be referred to available intervention [9]. Without access to rehabilitative services, children who experience developmental delays of disabilities struggle to achieve school readiness. School readiness is seen as a key predictor of school performance which is associated with adult employment and improved standards of living [10]. Cognitive disabilities often manifest themselves only during middle childhood (6–12 years) [11]. Research has shown that during this period, substantial changes can still occur as development is very malleable [10]. Hence, rehabilitation services that improve school readiness and performance are well suited to this phase of life.

School readiness occurs on three levels namely at the level of the child, their family, and the school environment [12]. “Child school readiness” refers to a specific level of language, cognition, physical-motor, and affective-social development that enables the learner to adapt easily, effectively, and without emotional disturbance within a formal teaching programme [10, 12, 13]. It is strongly associated with neurocognitive functions and behavioural attributes [10]. Children with cognitive disabilities may therefore need specific rehabilitative support to achieve school readiness. For this purpose, rehabilitative approaches such as sensory integration, psychomotricity, speech and occupational therapy, or psycho-educational interventions have been developed over the last decades [14–16]. Living with chronic HIV is a twenty-first century achievement in Africa, and rehabilitation services still need to be integrated with paediatric HIV care now that these children survive and live well with HIV. Interventions also need to be integrated into families and schools in order to adapt the child’s learning and play environments.

Despite the need for rehabilitation, a recent scoping review focusing on sub-Saharan Africa revealed lack of access to rehabilitation services for and appropriate research on children receiving paediatric HIV care [17]. The need for developing and establishing a platform combining rehabilitation and chronic paediatric care such as HIV care is highlighted [18, 19].

Access to rehabilitation services is viewed to be a function of the shortage of services (identification, referral and treatment, staff) and a lack of caregiver knowledge (regarding disability and rehabilitation) and resources (financial, physical) [12, 19]. In the context of HIV and AIDS, scholars have also highlighted the lack of integration between HIV and rehabilitation services [13, 20]. An integrated HIV and rehabilitative service that is feasible, acceptable, and practical for service users and providers is needed in order to overcome the multidimensional challenges associated with this group of children.

Novel rehabilitation methods have recently been collated into an e-module for rehabilitation professionals in the region [21]. However, we have little understanding of the acceptability, feasibility, and effectiveness of these interventions or how they can be integrated into existing HIV services. Isolated research has shown some promise in providing home stimulation programmes for young children (0–5) living with HIV [6]. Our recent study also indicated that a data management-driven system provides great support for the integration of rehabilitation services and medical care [20]. For the purposes of neurocognitive development, therapy approaches such as sensory integration and psychomotricity hold great potential and can improve school readiness during middle childhood [14–16]. In addition, different service delivery modes have been tested and also implemented outside the context of HIV [20, 22]. This provides examples of how rehabilitation can be adapted for resource-poor settings (e.g. WHO Community-Based Rehabilitation) [23]. Understanding how these novel approaches can best be utilised and integrated is critical.

Similar to other settings [10], an NGO at our proposed study site has applied a care delivery approach using task-shifting and peer support (caregiver-child group interventions). This approach includes an innovative service delivery mode, which trains local caregivers as physiotherapy assistants and teaches parents to be part of the child’s rehabilitation process. This delivery mode needs to be integrated with paediatric HIV care and for this innovative data management approaches that improve identification; referral and linkage to care can be used. Combining these innovations will likely provide a platform to develop a feasible, integrated rehabilitation and paediatric HIV care model [20].

**Fig. 1:**
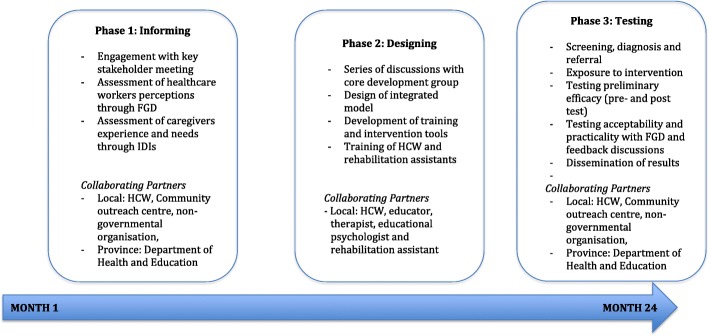
Mapping of the study process

The proposed study will design and test this integrated model in terms of its feasibility (acceptability, practicality, potential efficacy) from the caregiver and service delivery points of view throughout all stages of rehabilitation (assessment, referral, and interventions) (Fig. 1). It will feed into an existing research programme which has developed an evidence-based integrated model of rehabilitation services for adults living with HIV at the study site [18, 24–26] and which identified the need for rehabilitation of children living with HIV. It will provide the study site with an evidence-based integrated model that can be applied beyond the research project’s lifetime.

This study aims to inform, design, and determine the feasibility of an integrated rehabilitation and paediatric HIV model (initially targeting children living with HIV and disabilities between 5 and 10 years).

## Methods

The study builds on an existing research programme using an Integrated Learning in Action (ILA) approach [18, 20]. It will access participants through a public healthcare facility in a semi-rural area of KwaZulu-Natal, South Africa. The antenatal data at this site suggests close to 50% HIV prevalence rates, and healthcare workers have highlighted the need to address disabilities and schooling in children living with HIV. The study aims to develop this integrated model through:

1. Identifying factors (individual, institutional, community) determine acceptability and practicality of integrating rehabilitation into paediatric HIV from the perspective of (phase 1):
  - Healthcare workers when delivering paediatric HIV care or rehabilitation,
  - Caregivers of girls and boys living with HIV who are seeking services for their child’s HIV-related disability?
2. Designing the optimal design for a model integrating rehabilitation and paediatric HIV considering technological advances, evidence, acceptability, and practicality for the study site (phase 2) and
3. Establishing the feasibility of the integrated model with regards to (phase 3):
  - Preliminary efficacy of the integrated model on school readiness and disability-related outcomes amongst boys and girls with paediatric HIV (3–6 months pilot),
  - Acceptability and practicality of the integrated model from a caregiver and healthcare worker perspective?

### Phase 1: Informing the integrated model (6 months)

In phase 1.a., we will conduct multiple focus group discussions (FGDs) with 24–30 key stakeholders from the multidisciplinary healthcare team and community and short informative interviews to understand stakeholders’ perceptions of important factors to be considered when designing an integrated model. We will recruit participants using maximum variation sampling at the study site, selecting between five to eight health professionals, NGO representatives, and other key stakeholders.

#### Eligibility criteria for FGD

- Being part of the multidisciplinary healthcare team at the study site or their associated NGOs.
- Older than 18 years and working at the health facility or NGO for more than 6 months

The focus group discussions will be conducted by a facilitator trained in qualitative methods as well as a fellow researcher who will record non-verbal cues and facial expression. An experienced qualitative researcher will be present to moderate and oversee the process.

In phase 1.b., we will use in-depth interviews with 20 caregivers of children with cognitive disabilities. We will recruit participants purposively during their routine visit to the paediatric clinic. Eligibility will involve initial screening with The Washington Group Extended Set on Functioning (WG ES-F) [27]. Those children who screen positively will be assessed using the Wechsler Intelligence Scale for Children (WISC) by a psychologist (initially). The WISC is a widely, and locally, applied intelligence test that does not require reading or writing. It measures verbal comprehension, visual spatial perception, fluid reasoning, working memory, attention, and processing speed. This scale will identify children who have lower cognitive functions in one or more of these areas [28].

#### Eligibility criteria for IDIs

- Caregivers of children living with HIV, 5–10 years old in the phase of transition to school (preschool or grades 1–2) who are patients of the study setting.
- Children need to present with neurocognitive disabilities (Wechsler IQ of 90 and below) or developmental delays in one or more cognitive areas
- Caregivers who are not in an acute stage of disease (e.g. TB)

Recruited participants will be invited to interviews. These focus on the caregivers’ experiences with their children’s disabilities and their perceptions of the factors to be considered when integrating rehabilitation and paediatric HIV. All interviews will be conducted by a researcher trained in qualitative methods together with a moderator for the purposes of methodological rigour. The interviews will be recorded and transcribed verbatim. We will use conventional content analysis to understand the data as per Denyer and Tranfield [29].

### Phase 2: Designing the integrated model (4 months)

Through the ILA approach [18, 20], we will discuss the findings in phase 1 through a series of presentations and discussions with the core health team and caregivers. These will include reflections on factors regarding acceptability and practicality, identification of the best care delivery model (e.g. caregiver-child groups with rehabilitation assistant), linkage to care (screening tools/referral/data management systems), target group, and rehabilitation methods. As women are expected to be the primary caregivers, including many single parents, the design of the integrated model will take cognizance of possible gender biases.

Based on discussions, existing literature, and intervention material, we will design a model for integrated services including the data management system, consensus on appropriate screening tools such as the Parents’ Evaluation of Developmental Status [30], and diagnostic process; develop tools for the training of rehabilitation assistants; and adapt the space for the delivery of the pilot intervention. This entails training the paediatric nurses with disability screening tools and local rehabilitation assistants with the adapted intervention approach.

### Phase 3: Testing and formally evaluating the model (14 months)

In this phase, we will pilot and formally evaluate the feasibility of the integrated model (preliminary efficacy, acceptability, and practicality).

In phase 3.a., we will recruit at least 60 caregivers, and their children living with HIV and disability (predominately neurocognitive) will be recruited into the study and randomly allocated to intervention and control groups (control standard care, later crossed over). The sample size is based on current recommendations and practice of feasibility studies [31–34]. Participants will be identified during their routine visit using the Washington Group Extended Set on Functioning (WG ES-F) [27] and diagnostic tools (WISC) [28] and cut-off points as in phase 1.b.

#### Eligibility criteria for caregivers and children in intervention and control

The same as in phase 1.b., caregiver and child want to participate in the intervention.

A pre- and post-intervention evaluation will test the preliminary efficacy to achieve school readiness (using the Aptitude Test for School Beginners) [35]. Additional psychometric measures will be taken to assess cognitive functioning (Draw-a-Person test, working memory digit span), perception and social interaction between parents and children (the parent-child interaction questionnaire) [35, 36] and paediatric outcome measures. We will also use the Child Status [12] and a parent school readiness self-reported questionnaire [12] to monitor enrolment, correct class placement, and performance.

The intervention will target two different areas to improve school readiness over a 6-month period: firstly, each child’s cognitive development and skills (psychomotricity-based therapy sessions) and secondly, the parents’ learning environment/routine at home (parents as therapy assistants). The data analysis will include descriptive statistics and ANOVA/ANCOVA to analyse intervention effects.

In phase 3.b., we will conduct FGDs with the caregivers from phase 3.a. and healthcare workers to evaluate the acceptability and practicality of the integrated model. The FGDs will prompt experiences with the screening process, data management system, intervention, and preparation for school as well as perceptions around feasibility. The discussions will help build consensus about the elements needed for an integrated model. Discussions will be recorded, transcribed, and analysed using guided content analysis [29] focusing on perceived efficacy, acceptability, and practicality.

Another feedback meeting at the study site will disseminate results and allow for discussion of the adapted version of the integrated model. Furthermore, we are participating regularly in government meetings where the results from this study will also be presented.

## Discussion

The current transformation of South Africa’s health system towards universal health coverage is a critical phase towards better health and well-being for all. Within this process, the country has taken a crucial step with the design of its Framework and Strategy for Disability and Rehabilitation Services in South Africa (2015–2020), which embeds “rehabilitation as an important component in the continuum of care and essential to a good quality of life and increased life expectancy” (p. 5 [37]). The contemporary transformation of health services is seen as an opportune moment to reconfigure rehabilitation as an integral part of all health services, in particular those that are health priorities such as HIV and AIDS.

However, the current system has many challenges in particular; we lack feasible models that facilitate integration of rehabilitation and chronic care such as HIV care. Within the last years, a number of good practice case studies have been identified for adult care that show great potential to facilitate integration rehabilitation into current care [16]. However, for children living with chronic diseases such as HIV in Africa, we have no scientific evidence that provides information on acceptable and feasible approaches for integrated rehabilitation. Hence, the proposed study will provide important information on how integration can be facilitated in current HIV-paediatric care as well as what elements need to be considered when preparing for a full evaluation of such a model such as a randomised control trial (RCT). The study will provide information for a future RCT such as recruitment strategies, sample size, effect size, and choice of feasible screening tools.

## Abbreviations

ART: Anti-retroviral therapy
BREC: Biomedical Research Ethics Committee
HIV: Human immunodeficiency virus
IDI: In-depth interviews
KZN: KwaZulu-Natal
NHI: National Health Insurance
PHC: Primary Health Care
SA: South Africa
WISC: Wechsler Intelligence Scale for Children
